# Examining clinician choice to follow-up (or not) on automated notifications of medication non-adherence by clinical decision support systems

**DOI:** 10.1186/s12911-022-02091-2

**Published:** 2023-01-30

**Authors:** Dan Thorpe, Jörg Strobel, Niranjan Bidargaddi

**Affiliations:** 1 Digital Health Research Lab, College of Medicine and Public Health, Flinders University, Adelaide, SA 5042 Australia; 2 Barossa Hills Fleurieu Local Health Network, SA Health, 29 North St, Tarrawatta (Angaston), Peramangk Country, Adelaide, SA 5353 Australia

**Keywords:** Clinical decision support systems (CDSS), Digital psychiatry, Proactive care, Interaction design, Embedded mixed-methods study design

## Abstract

**Background:**

Maintaining medication adherence can be challenging for people living with mental ill-health. Clinical decision support systems (CDSS) based on automated detection of problematic patterns in Electronic Health Records (EHRs) have the potential to enable early intervention into non-adherence events (“flags”) through suggesting evidence-based courses of action. However, extant literature shows multiple barriers—perceived lack of benefit in following up low-risk cases, veracity of data, human-centric design concerns, etc.—to clinician follow-up in real-world settings. This study examined patterns in clinician decision making behaviour related to follow-up of non-adherence prompts within a community mental health clinic.

**Methods:**

The prompts for follow-up, and the recording of clinician responses, were enabled by CDSS software (AI^2^). De-identified clinician notes recorded after reviewing a prompt were analysed using a thematic synthesis approach—starting with descriptions of clinician comments, then sorting into analytical themes related to design and, in parallel, a priori categories describing follow-up behaviours. Hypotheses derived from the literature about the follow-up categories’ relationships with client and medication-subtype characteristics were tested.

**Results:**

The majority of clients were Not Followed-up (n = 260; 78%; Followed-up: n = 71; 22%). The analytical themes emerging from the decision notes suggested contextual factors—the clients’ environment, their clinical relationships, and medical needs—mediated how clinicians interacted with the CDSS flags. Significant differences were found between medication subtypes and follow-up, with Anti-depressants less likely to be followed up than Anti-Psychotics and Anxiolytics (χ^2^ = 35.196, 44.825; *p* < 0.001; v = 0.389, 0.499); and between the time taken to action Followed-up_0_ and Not-followed up_1_ flags (M_0_ = 31.78; M_1_ = 45.55; U = 12,119; *p* < 0.001; η^2^ = .05).

**Conclusion:**

These analyses encourage actively incorporating the input of consumers and carers, non-EHR data streams, and better incorporation of data from parallel health systems and other clinicians into CDSS designs to encourage follow-up.

**Supplementary Information:**

The online version contains supplementary material available at 10.1186/s12911-022-02091-2.

## Background

Medication adherence—that is, a person consistently and correctly following a mutually agreed upon, collaborative plan made with their clinician for using medication to manage a condition [1]—is core to successfully managing chronic conditions across a variety of populations [2–7].

Medications are often an essential component of plans to reduce the risk of relapse for many people diagnosed with complex mental illnesses; as such, they are core parts of lives of many people living with schizophrenia [3, 8], bipolar disorder [9, 10], and major depressive disorder [11]. Investigating means of promoting and maintaining adherence to these medications is, therefore, a priority in clinical mental health research [4, 5]. Indeed, between 1 in 2 and 1 in 4 people who take antipsychotics become non-adherent during the course of their illness, and half of people diagnosed with bipolar are estimated to become non-adherent to their medications at least once during the long-term course of their illness [10, 12–14]. This can occur for a variety of reasons. Supporting one perspective, a systematic review exploring the identification of “potentially modifiable” (ie., feasible bases for developing interventions) reasons for non-adherence to anti-psychotics identified poor insight, substance abuse, negative attitudes toward medication and side-effects—among others [6]. However, other studies have argued that non-adherence is not solved by policing or sidelining concerns and beliefs about medication [10]. First, people—regardless of the perceived severity of their illnesses—can and do discontinue medication for valid and well thought out reasons [15]. Additionally, the generalisation of population-level outcomes used to justify common pharmacotherapeutic treatments to individuals may delay recovery for some [16]. Regardless of perspective, core to protecting long-term mental and physical health outcomes of people with complex mental illnesses is ongoing support from their clinicians and community [16–18]; and, where non-adherence is identified as problematic, that support is provided in a context that encourages candour, trust, and transparency from and between all parties [15, 19, 20].

However, complexity of illness is not the sole factor predicting non-adherence, nor do “lower-risk” diagnoses and psychiatric medications necessarily result in less risk to people’s health if they abruptly discontinue; namely antidepressants [7, 13, 17, 18, 21, 22], which are prescribed to an increasing share of the population. Indeed, two Selective Seratonin Reuptake Inhibitors (SSRIS)—Escitalopram (5.47 million prescriptions) and Sertraline (5.12 million prescriptions)—appeared in the top ten drugs by prescription count in Australia in 2020–21 [23]. In a naturalistic study of a sample from the United States of America, it was found that — when participants were asked about their medication adherence in the year prior to their participation in the study—22% of anti-depressant users had discontinued antidepressants without clinician advice or approval [13]. Another study found that the rate of discontinuation also increases over time in anti-depressant users, showing adherence rates of only 37.6% at 3 months, and 18.9% at six months [21]. These data, alone, are not necessarily cause for concern—but are important to keep in mind when contrasting the relative low-risk assigned to anti-depressant discontinuation effects in policy [17] with recent literature [7, 18, 24]. For example, a recent systematic review found 56% of people discontinuing anti-depressants experienced withdrawal effects, 46% of whom described them as severe and longer than outlined in current UK and US guidelines [18]. These potentially urgent grounds for intervention are complicated further by these events often coinciding with the termination of the clinical relationship [22], making important clinical scaffolds for discontinuation—identifying facilitators for successful discontinuation, co-designing a personalised plan with the person discontinuing, relapse planning, involving a family member or trusted other, and setting up continuity of care provision with the person discontinuing [25, 26]—a virtual impossibility.

### Clinical decision support systems

Given the adverse consequences for many people who discontinue their medication [26–28], early intervention is key [29]. Digital tools offer potential means for health services to proactively provide care and support in these contexts. Clinical decision support systems (CDSS) are an example of such a tool. CDSS first curate data from sources that can include but are not limited to sources such as: electronic health records/clinical information systems [30, 31], sensing technologies ranging from consumer products like mattress sensors to therapeutic devices like continuous glucose monitoring systems [32, 33], SMS surveys of clients [34], and self-monitoring apps [35, 36], amongst others. These data are then presented to users in a manner that informs a clinical decision—either through algorithmic interpretation, using decision rules based on a pre-existing knowledgebase to suggest a course of action [37], or simply through a more intuitive presentation of the raw data [38]. These systems assist in making sense of sometimes vast data, transforming individuals’ patterns in service use, medication adherence, and in some cases elements of their day-to-day life to a form more immediately legible to clinicians [39]. This making-legible of raw data in turn enables the development and delivery of interventions with, theoretically, highly granular levels of client-specificity that would not be feasibly achievable at scale and within the time constraints of a human agent; both augmenting human delivered support at the point of care and potentially enabling tailored, automated follow-up independent of traditional in person contact with a clinician [37]. In the context of medication adherence this latter consideration is particularly important, with multiple authors emphasising the lack of a “one-size-fits-all” intervention, and need to tailor any approach on a client-by-client basis using nuanced, ecological insights into their lives as a basis [19, 20, 40]. Finally, where these data streams are real-time or close-to-real time, clinical teams can be enabled to monitor to evaluate the success of these interventions and respond to any changes in the client’s state, should they arise, in a proactive and timely manner.

While the systems described above certainly have the *potential* to reduce the burden of relapse and deterioration of mental health associated with non-adherence on clients and services, the evidence in the literature is ambivalent [31, 41–45]. Indeed, reviews have consistently noted the low quality of evidence, risk of bias, and need for further research in this field [42, 45, 46]. Regardless, CDSS are already in use for the management of some high-risk medications within mental health services in Australia—for example, in clozapine management to enable proactive intervention into non-adherence triggered relapse and early detection of adverse events and side effects [47, 48]. Authors of a recent, 5-year database study of antipsychotic utilisation and persistence in a large Australian sample conclude that oral Clozapine’s significant persistence in comparison to both other oral anti-psychotics *and* Long-Acting Injectable antipsychotics could be attributable not only to efficacy but intensity of follow-up [49].

### Contextual barriers to decision support

Thorough and multifaceted work on a variety of fronts is required when designing these tools and their associated interventions. Proficiently executing facets of CDSS development like user interfaces, user experience, and balancing alert fatigue with under-prompting are rightly identified by many as important for success [46, 50–52]. However, equally important is the manner in which a CDSS integrates into both the workflows and self-perceptions of its future users [53, 54].

Regarding the latter, clinicians have shown resistance to the use of algorithms in healthcare—both “analogue” in the early days of guideline based care [55, 56], and digital [41, 53]. This resistance stems from clinicians’ strengths in adaptive expertise [57], but can also limit acceptance of other experts’ opinions [53, 55]. For researchers committed to actualising the potential for CDSS to enable proactive and evidence based care, knowledge of these complexities and their effect on behaviour is crucial to success but can be elusive—emerging more prominently in the naturalistic, day-to-day work performed by clinicians than under controlled circumstances [41, 49, 53, 56, 58]. This is important to consider in the context of study designs for evaluating CDSS. The results of Randomised Control Trials (RCTs), where clinician actions are strictly protocolisation, may not fully reflect the behavioural and practical realities of clinical practice [41, 58, 59]. Time limited, protocolised workflows introduce an artificial “order” to clinical work for trial durations, resulting in masking the biases and work practices that may occur automatically, as a result of external pressures, or for any other reason in day-to-day practice outside of the trial [51, 52, 60].

Outside of these external factors, it is important to note that—for many clinicians—it is preferable for a variety of reasons to rely on their experience and judgments rather than that of a system [53]. These factors can severely impact the efficacy of CDSS interventions, regardless of trial and software design quality [41]. As such, it is important not only to understand at a systemic and organisational cultural level why CDSS implementations face challenges, but also to develop methods and design tools that can collect data from which we can establish how and why individual clinicians make decisions using these systems, naturalistically and in the moment [53, 61].

### The current study

This study presents results from the pilot of a real-time, CDSS-integrated technique to gather data showing *how*, *why*, and *when* clinicians acted on automated medication non-adherence flags, aiming to: (1) Describe patterns of follow-up behaviour within these non-adherence data; (2) Identify areas for design intervention within CDSS; and (3) Identify any relationship between client and medication subtype characteristics and the likelihood of follow-up. These flags were generated by a CDSS—Actionable Intime Insights (AI^2^), a web-based medication and appointment adherence CDSS—using data from the Australian Medicare claims databases [29, 30, 39]. Free-text justifications of decisions to follow-up or not follow-up were input by clinicians throughout the trial, and extracted in parallel with other flag metadata, including medication subtype, client ID, days taken to action the flag.

Descriptive data outlining decision behaviours—alongside medication-subtype and client characteristics—were extracted from the raw AI^2^ flags. These data were then analysed and synthesised using parallel qualitative and mixed methods [62–65]: first, through thematic synthesis, with analytical themes generated through qualitative synthesis of the descriptive codes [63]; second, hypothesised relationships between medication-subtype and client characteristics with follow-up were tested using inferential statistical techniques [62, 65]. The discussion presents a summary and synthesis of these findings, focussing on the implications for future CDSS design and implementation studies.

## Methods

### Ethics and consent to participate

The AI^2^ study protocol was approved by the Southern Adelaide Local Health Network Clinical Research Ethics Committee (AK03478) and published prospectively [29]. An informed consent was obtained from clinicians participating in this study. As per the My Health Records Act (2012) legislation, all consenting clinicians have rights to use AI^2^ CDSS to access health records of patients for the purposes of care provision without requiring explicit consent. The extraction and analysis of de-identified AI^2^ CDSS data for this study was in accordance with the guidelines approved by the ethics committee.

### Abridged primary trial procedure

As this study analyses data from the AI^2^ implementation, relevant details of the design of that study have been included here to contextualise this analysis.

### Participants

#### Clinicians

Two clinical monitors used the AI^2^ decision support software prospectively with 354 clients under their care managment, choosing to follow-up or not follow-up on flags as they were raised:

1. A Social Worker and Team Leader within the service; and
2. A Senior Consulting Psychiatrist, Author JS

Clients seen by clinical monitors had: (1) attended the community mental health clinic associated with this study at least once before in the six months prior to the study; (2) prescribed medication for their mental health condition; (3) had a My Health Record (Australia’s national digital health record); and (4) were registered in the clinic’s client information systems and subsequently enrolled in AI^2^ and monitored for non-adherence between 1 July 2019 and 28 February 2020.

### Materials: AI^2^

**Fig. 1:**
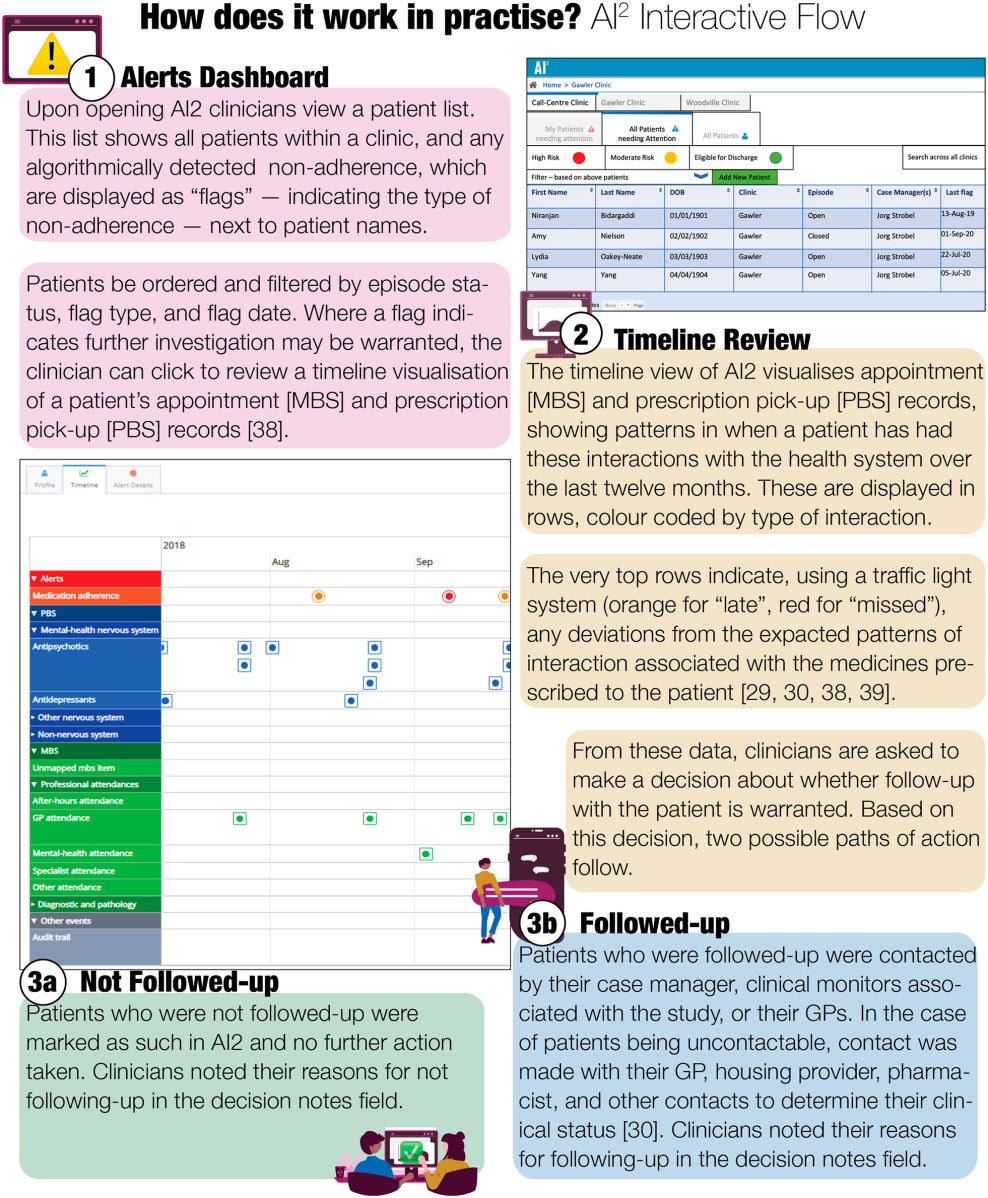
Flow diagram demonstrating interactive patterns with AI^2^ alerts

Figure 1 illustrates the interactive flow with AI^2^ experienced by clinicians in the trial in more detail; more detail about the software and primary trial is reported elsewhere [29, 30, 38, 39, 66]. The pilot trial studied the implementation and impact of AI^2^ by incorporating it into the usual provision of care at the pilot site. As such, the protocol included no specifications about when to follow up, worked within the team structures and staffing resources available at the site, and within the day-to-day working norms of the clinical monitors [29, 30]. This approach was chosen to allow for insights closer to the naturalistic conditions facing implementations in practice.

The procedure for clinicians using AI^2^ involves following steps:

1. Reviewing non-adherence flags on the dashboard (Fig. 2).

**Fig. 2:**
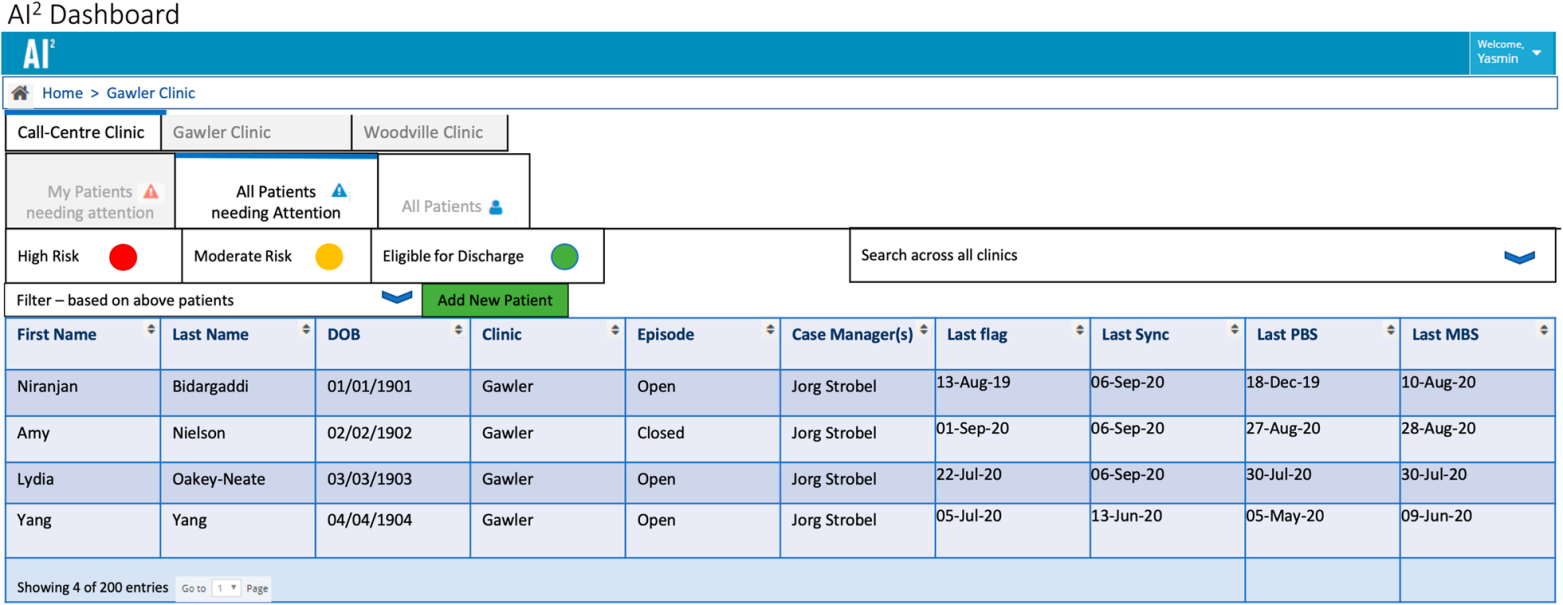
AI^2^ Alerts Dashboard using primary trial investigator names for example purposes
2. If a non-adherence flag, in the reviewing clinician’s judgment, warrants further investigation they examine the client’s records—including:
  - The timeline within AI^2^ (Fig. 3), which visualises patterns in medication pick-up and appointment attendance data collected in near real-time [38];

**Fig. 3:**
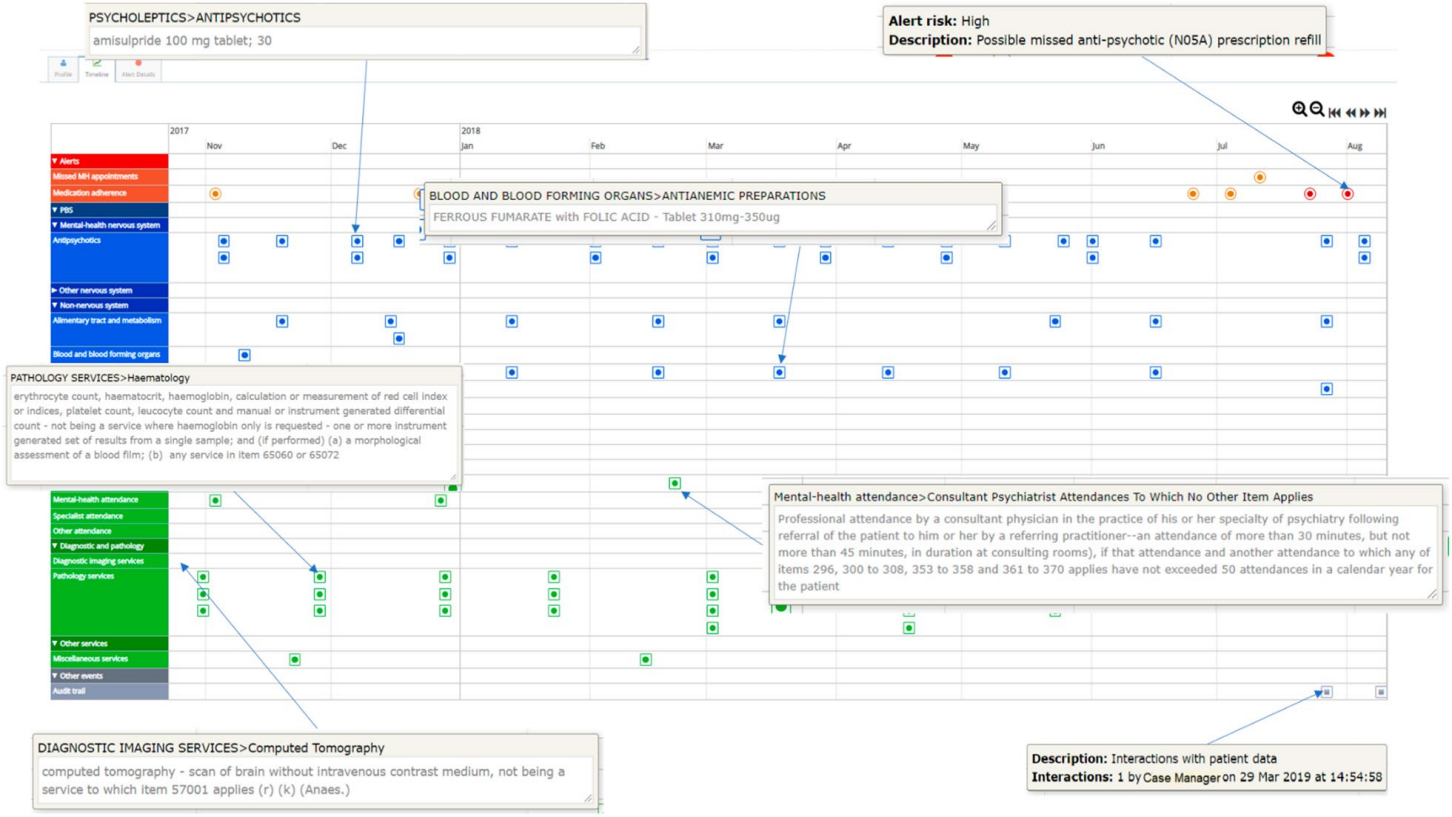
Timeline Detail, with example “pop-up” information-boxes associated with clicking on flags, MBS, and PBS events shown
  - Relevant data in the implementing service’s clinical information system;
3. Based on these data, the clinician then chooses to follow up (or not) on the flag
4. Finally, they record this choice in AI^2^, and provide brief comments in the follow-up notes form (Fig. 4).

Following this, specific to the trial site, the clinician emailed a coordinating Registered Nurse appointed to oversee the follow-up and data entry. Clients who were followed-up were contacted by their case manager, clinical monitors associated with the study, or their GPs. In the case of clients being uncontactable, contact was made with their GP, housing provider, pharmacist, and other contacts to determine their clinical status [30].

### The current study

#### Data collection technique: theoretical background and design

The objective of the AI^2^ pilot implementation study was to establish a more comprehensive, naturalistic evidence base from which the multi-faceted requirements of full-scale implementations of CDSS can be elucidated [29]. Extracting patterns in *clinician*-user behaviour was identified as a key adjunct to the primary quantitative analysis in answering the research questions of the trial—with the aim to iteratively improve the fit the intervention to clinician workflows. [67]. However, these naturalistic conditions also necessitated careful design. Researchers needed to balance adversely impacting clinical workflows with encouraging action on flags. To the former, researchers risked either potentially discouraging use of the tool, or—conversely—creating an artificial level of adherence to a protocolised version of our imagined usage of the tool that would result in key implementation barriers in the day-to-day of health services being missed. To the latter, the researchers equally did not want to inadvertently fail to achieve the primary aim of the trial through this naturalism—to encourage action of flags and understand the effect of this proactive care on health services and individual clients.

This design problem is common to guidelines engagement interventions more broadly, and solutions to encourage adherence without compromising workflows or naturalism have proven less than intuitive. For example, while integration with clinical information systems (CIS) seems an intuitive option, this work is technically difficult [37]. Additionally, CIS are often misused—in a benign way—to sidestep time-consuming or poorly designed features. An example of this can be found in Förberg and Colleagues’ CIS-integrated decision support study, where nurse participants’ use of a more generic template to log data—rather than the template intended for use to record outcomes for the clinical action of interest—inadvertently resulted in participants missing the guideline reminders that formed the core of the intervention [41]. Within and outside of CIS-based interventions factors such as alert fatigue, and a reduction in perceived “seriousness” of alerts in the context of an overwhelming amount of data can also challenge designers and implementation scientists in this space [41, 68–70]. One method with a more established history of success in addressing tendencies to avoid or dismiss advice is designing systems to require entering a reason when overriding advice, which one systematic review found resulted in higher adherence to CDSS advice [68]. However, the authors note that highly insistent systems can either frustrate clinicians into underuse or encourage un-critical acceptance of automatically generated advice [68]. Additionally, this review also note that systems using structured data collection techniques can inadvertently bias responses through priming [68].

**Fig. 4:**
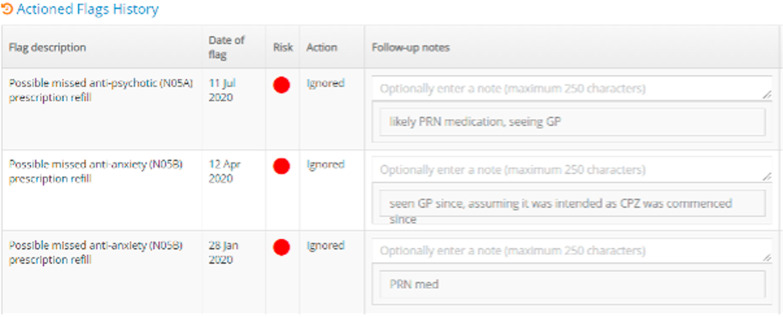
AI^2^ Alert actioning and decision data collection interface

To balance these concerns, we settled on a simple data-collection technique—a non-compulsory, free text field at the time of alert actioning—in which we asked clinicians to briefly note their reasoning behind following-up or not following-up on an alert (Fig. 4). This was operationalised within a concurrent-nested mixed methods design, collecting these data in parallel to the primary non-adherence flag metadata—such as medication subtype, the time lag for actioning the flag, and so on—of interest to the primary AI^2^ implementation study.

We begin with the extraction of descriptive data—inductive and descriptive coding of the decision notes, descriptive statistics, and sorting of these combined data into a priori categories (Followed-Up, or Not Followed-up)—from the raw flags. This was followed by parallel analyses—beginning with Thematic Synthesis of the descriptive codes exploring design insights, and followed by mixed-methods hypothesis testing exploring relationships between client and medication-subtype characteristics and follow-up behaviour. Table 1 outlines the aims, objectives, hypotheses (where appropriate), and outcomes (including measures and tests, where appropriate) of this study (Table 2).

**Table 1:** Aims, objectives and hypotheses, and outcomes

Aims	Objectives/hypotheses	Outcomes
Phase One: Feasibility and Utility Analysis (Mixed Methods Anaylsis, Framework Method)		
1.1) Establish the feasibility and utility of the data collection design	1.1.1) Evaluate compliance with the note-collection system	1.1.1) Evidence of feasibility of this data collection design within practice
	1.1.2) Report any difficulties that arose in descriptive qualitative analysis process	1.1.2) Evidence of the collected data’s utility for qualitative insights into decision behaviour
1.2) Describe patterns of follow-up behaviour within the non-adherence data, establishing the feasibility and utility of AI2 to enable follow-up	1.2.1) Report on repeated follow-up decision behaviours in the data	1.2.1) Inductively derived descriptive codes, describing repeated patterns of decision behaviour
	1.2.2) Use these codes to group notes into behavioural categories within the a priori framework of follow-up behaviours	1.2.2) Deductive categorization of and, therefore, generation of frequency data for code occurrence within categories of follow-up using a framework method approach
	1.2.3) Describe frequency of behavioural patterns within different metadata derived categories of interest	1.2.3.1) Between-medication subtypes and follow-up status descriptive statistics 1.2.3.2) Within-patient, between-follow-up status descriptive statistics
Phase Two: Generation of Design Insights (Qualitative Analysis, Thematic Synthesis)		
2) Explore emergent interaction behaviours with the non-adherence data beyond the categories of followed-up and not followed-up	2.1) Explore barriers to and facilitators for follow-up behaviours within AI^2^	2.1) Analytical themes going *beyond* the raw data and generating new categories for intervention and experimentation
Phase Three: Preliminary evaluation the impact of medication and patient-level characteristics on follow-up (Mixed Methods Analysis, Framework Method)		
3) Addressing the problem of establishing—quantitatively—whether CDSS impacted clinician choice using data from Phase One	Hypothesis 1 (H1): The number of flagged patients followed-up will differ significantly between medication subtypes	*Test(s):* Chi-squared (χ^2^) test of homogeneity (Cramér’s *v* to indicate effect size) to confirm variance in distribution of follow-up status between-drugs. Pair-wise Fisher’s Exact tests of independence (χ^2^ statistic and Cramér’s *v* to indicate effect size) to explore significance of difference between individual drug types *Assumptions,* χ^2^*:* a) Independence of observations b) No more than 20% of cells have an expected frequency of < 5, no cell has an expected frequency < 1 c) χ^2^ < critical value for the relevant degrees of freedom [88–90] *Assumptions, Fisher’s Exact Test:* d) Independence of observations e) Fixed column totals, *however*, also appropriate where column totals are not fixed should cell sizes be too small for a χ^2^ test [92] *Reported statistics:* χ^2^ statistic, expected counts per cell, actual counts per cell, p value, Cramér’s *v*
	Hypothesis 2 (H2): The time taken by clinicians to action flags will differ significantly between medication subtypes	*Test(s)*: It is anticipated that these data will not be normally distributed; this assumption will be tested with Shapiro–Wilk tests Kruskal–Wallis H Test, η^2^ for effect size *Assumptions:* a) Independence of observations b) Cell size > 5 c) Continuous distribution [89] Should the null hypothesis be rejected, a squared ranks test — exploring homo/heterogeneity of variances between samples will be conducted [89–91] *Reported statistics: H* statistic, count per cell, *p* value, η^2^ statistic
	Hypothesis 3 (H3): There will be a significant difference in the time taken by clinicians to action flags between the two categories of follow-up	*Test(s)*: It is anticipated that these data will not be normally distributed; this assumption will be tested with Shapiro–Wilk tests Mann–Whitney U Test, η^2^ for effect size *Assumptions:* As per the Kruskall-Wallis H Test *Reported statistics: U* statistic, count per cell, *p* value, η^2^ statistic
	Hypothesis 4 (H4): In patients with mixed follow-up status on their flags, a monotonic time × event relationship will exist — with follow-up more likely to occur in this group as the number of flagged non-adherence events increases	*Test(s)*: Time × event (Cox proportional hazards) regression, log–log plots *Assumptions:* 1) Non-informative censoring; that is, individuals not participating in the study would have the same probability of experiencing follow-up as those in the study should they have participated 2) Hazard functions remain proportional (eg., if an individual—at baseline—is less likely to be follow-ed up than another individual, this should not change over time). Tested with log–log plots *Reported statistics:* Coefficient, standard error, hazard ratio, 95% CI, *p* value, log–log plots [93]

**Table 2:** Follow-up categories and descriptive codes × number of flags

N	Ref	Category/*Sub-Category*/descriptive decision code	Explanatory notes	Example codes
71	F	Followed up		
20	F.1	Adverse Outcomes		
1	F.1.1	Died		“Pt has died”
14	F.1.2	Hospital Admission	Different dispensing system, no data	“Recent hospital admission discharged 12/3. case manager confirmed current compliance”
5	F.1.3	Incarcerated	Different dispensing system, no data	“Given antipsychotic and oral meds in June. (so likely a delay in PBS data) This client is now in remand. Prison health service has been made aware of his medications. No longer residing in our catchment area.”
46	F.2	Confirmed adherence		
6	F.2.1	Ambiguous source of confirmation	Ie., source was not named in the notes	“Compliant with medication (Data incorrect collects scripts every month)”
5	F.2.2	Client Confirmed		“25/5/20 contact with client. confirmed compliance. no problems”
14	F.2.3	Clinician or Case Manager Confirmed		“Case manager: According to CPMS all medication has been dispensed for every month”
5	F.2.4	Clozapine	Monitored elsewhere in service	“R/v client file. P/c and 1:1 with Hyde and partners GP. Client presented for GP appt and collected script for clozapine on 10/08/2020. Nil concerns raised”
2	F.2.5	Depot records showing compliance		“Accumulating data from all sources client received depot as prescribed on the below dates”
4	F.2.6	Family confirmed		“His mother pick-ups the medication and deliver at his place”
13	F.3	Confirmed non-adherence		
1	F.3.1	Appointment booked to discuss compliance		“Awaiting appointment for follow up with information regarding compliance of medication”
2	F.3.2	Client lost to follow-up, case manager pursuing	Client disappeared, or disengaged	“Client whereabouts unknown. Case manager aware of non-compliance”
2	F.3.3	Client refused further intervention		“P/C to client, declined services. Fax sent to GP.”
7	F.3.4	Discontinuation confirmed on follow-up		“Ceased September 2020 was doing well then come unstuck and thought he needs to go back to the GP and re-commence taking it”
3	F.3.4.1	Ambiguous	Unclear who initiated/supported	“Intentional cessation”
1	F.3.4.2	Initiated with Medical professional support		“Spoke to client, stopped antipsychotic as recommended by [clinician]”
3	F.3.4.3	Patient initiated, unsupported		“Stopped taking medication as he had been on it for a long time (2 months) and it did not help.”
260	N	Not followed-up		
140	N.1	Likely to be Clinician Supported		
11	N.1.1	Changed Medication		“Paliperidone injection monthly changed to 3-monthly (TRINZA)”
1	N.1.1.1	Changed to depot		“Change to depot”
13	N.1.2	In residential care	Monitored by facility in Aus. context	“Lives in boarding house where medication is supervised”
79	N.1.3	Likely PRN	PRN = taken as needed	“PRN medication such as diazepam and oxacepan should not set triggers”
22	N.1.4	Script likely a short-term solution		“Was a once off script, regular GP visits”
15	N.1.5	Seen their GP, while		“Seen GP since, assuming it was intended as CPZ was commenced since”
13	N.1.5.1	Compliant with other medications		“Regular in everything else, likely per Gp”
2	N.1.5.2	Started a new medication		“GP seemingly trying different antidepressants”
*133*	*N.2*	Unclear without further investigation		
14	N.2.1	Assumed to be a trial		“As only once dispensed I assume that it was poorly tolerated”
68	N.2.2	GP visit regularity only source of data for assumption		“Several GP appts since- assuming intentioned”
42	N.2.3	Irregular prescription pickup pattern	Ie., assumed normal behaviour for pt	“Likely timing issue, he picked up the last repeat too early, long term very reliable”
9	N.2.4	Patient known to be non-compliant, not followed up		“Patient has a history of noncompliance with limited benefit of medication, as such likely real alert but no action taken before next scheduled review”
2	N.2.4.1	Patient has case manager	Unclear if C.M. was followed up	“Case managed, known non-compliance”

### Analysis plan

De-identified decision note and flag metadata from AI^2^ were analysed using NVivo R1 (QSR Software, 2021) and SPSS v.27 (IBM Corp, 2020). The data included 771 medication and appointment non-adherence flags across 304 clients. The trial occurred between 11th Jan 2020 and 14th of November 2020. Importantly, a client may have multiple flags on the same date based on different algorithms. On these occasions, the assessing clinician duplicated their notes across both flags, as they were actioned in the same manner at the same time in all cases. For the purposes of analysis, these duplicates were coded identically so as not to unbalance numbers of flags across categories.

#### Coder details and risk of bias

For the initial descriptive analysis of the codes this study utilised a single coder with clinical oversight—provided by Author JS, one of the clinical monitors in the trial—to ensure closeness of the analysis to the clinical context on which it reports [63]. While a single coder is not preferable in most qualitative approaches, there are mitigating circumstances in the case of this study. First, the small scale and reduced scope of this pilot analysis, and the focus on design insights of the findings tempers the potential generalisability of these findings *clinically*, mitigating the risks of publishing these data. Second, the relative simplicity and brevity of the qualitative data included for analysis in this study (examples are given within the results section of this paper) reduce the potential for different *categorical* interpretations of the flags. Third, authors JS and NB contributed to the generation and refinement of the more inferential, perspective-driven analytical themes—meaning the core generalisable design findings of this study represent consensus between multiple authors and mitigating single-coder bias.

Finally, the risk of bias associated with a single coder was also managed by engaging a researcher external to the project. Author DT, who conducted the analyses in this study, began work at Flinders after the cessation of the trial, has no prior relationships with any participants or clinicians involved in the trial, is not involved in the AI^2^ project, and his pay and role originate from an entirely separate project. His role in clinical mental health services—as a peer practitioner—is separate to that of both clinical monitors, but has also encompassed triage, assessment, and intervening in non-adherence.

#### Descriptive data analyses

Flags relating to medication non-adherence with clinical note data were included for anaylsis. First, research questions were set aside, and decision notes associated with medication non-adherence flags were inductively coded based on the behavioural justifications for follow-up they described [63]. These flags were then sorted into the deductive, a priori categories embedded in research question one and the study protocol [29]—Followed-up and Not Followed-up—and further subcategories inductively derived following a framework method approach [62, 65].

Following this, quantitative data, matched to the decision notes, were extracted from AI^2^. These data—flag ID, system-generated client ID, “flag raised” time stamp, “flag actioned” time stamp, medication subtype—were imported into IBM SPSS Statistics (version 27, 2021). Data analyses were conducted by author DT and reviewed by author NB. Time stamps were used to compute a days-to-action variable for each flag, providing the number of days before flags were actioned for each flag. Shapiro–Wilk Tests of Normality were used to determine the normality of the resulting distributions associated with these data. Descriptive statistics were produced through mixing the qualitatively derived framework method categories and flag metadata of interest.

#### Inferential analyses

The qualitative element of these analyses continued the thematic synthesis derived approach of initiated in the descriptive analyses through the generation of analytical themes—that is, inferential, generative, and exploratory themes that “go beyond” the implications of the raw data and identify sites for CDSS design intervention [63]. These themes were derived through both individual and consensus exploration of patterns between and within descriptive codes by the authors of the study. These insights, along with their respective descriptive code bases, were reported.

This work was augmented utilising a mixed-methods approach, mixing the qualitatively-derived Follow-Up categories with quantitative data derived from the metadata—relating to medication subtype, patterns in client adherence, and time taken to follow-up. Hypothesised relationships between these metadata and their impact of on clinicians’ follow-up behaviour were derived from the literature presented in the background to this paper; the nature of these relationships and how they will be tested is outlined in Table 1. This mixing is justified; indeed, combining these data provides a coherent integration of longitudinal and rich mixed data, augmenting the standalone quantitative and qualitative data. What constitutes “follow-up” is deeply contextual to both the type of clinician and service under investigation, as are the behaviours that inform these decisions. This mixed-methods approach flexibly allows for high-level comparisons (at the follow-up level) *and* nuanced exploration of variations in how services and clinicians conceptualise and operationalise these constructs. This means this approach is, ultimately, reusable—allowing for replications that reflect the nuances of new contexts and clinicians while still accommodating comparisons and syntheses between contexts.

### Reporting guidelines

This paper reports data conformant with APA-JARS MMARS standards [71, 72]. See Additional file 1: Appendix 1 for an annotated copy of these guidelines with section references for relevant data.

## Results

### Descriptive analyses

Following these initial analyses, 331 flags for 179 clients met the inclusion criteria for further analysis. Clinical decision related notes fell into two top-level categories: Followed-up (n = 71; 22%) and Not Followed-up (n = 260; 78%). The Followed-up category was further subcategorised into: (1) Adverse outcomes (n = 20); (2) Confirmed evidence of non-adherence (n = 12); and (3) Confirmed adherence (n = 46), either from the client, their family or their GP. The Not Followed-up category was further subcategorised into: (4) Unclear without further investigation (n = 133), where there was evidence of non-adherence, action was deemed unnecessary by the clinician, and the clinical notes did not specify or minimally specified the evidence for their decision; and (5) Likely to be Clinician Supported (n = 140), where there were multiple data-points supporting the hypothesis that the client was being well-managed. Subcategories, frequencies, and example codes for this analysis are provided in Table 3.

**Table 3:** Flags × medication type

Prescription type	N. Flags	%	Followed-up	Not followed-up
Mood stabilisers	21	6	5	16
Anti-Parkinsonians	6	2	1	5
Anti-psychotics	102	31	37	65
Anxiolytics	50	15	2	48
Sedatives	5	2	0	5
Anti-depressants	130	39	23	107
Psychostimulants	12	4	2	10
Nervous system drugs	5	2	1	4
Totals	331		71	260

### Qualitative analysis: design insights from thematic synthesis of decisions notes

Three major themes, two with sub-themes, were identified across follow-up categories; Table 4 shows associated descriptive codes and case frequencies for each.

**Table 4:** Analytical Themes and Descriptive Codes × Number of Flags

N	Ref	Analytical Theme*/Analytical Sub-theme/* Descriptive codes	Explanatory Note		Example code
78	A.1	Non-health record data is key to encouraging follow-up			
39	*A.1.1*	*Data gathered from other record keeping systems*			
14	F.1.2	Hospital Admission	Different dispensing system, no data		*“Recent hospital admission discharged [DATE] case manager confirmed current compliance”*
5	F.1.3	Incarcerated	Different dispensing system, no data		*“Given antipsychotic and oral meds in June. (so likely a delay in PBS data) This client is now in remand. Prison health service has been made aware of medications. No longer residing in our catchment area.”*
5	F.2.4	Clozapine	Monitored elsewhere in service		*“R/v client file. P/c and 1:1 with partners GP. Client presented for GP appt and collected script for clozapine on [DATE]. Nil concerns raised”*
2	F.2.5	Depot records showing compliance			*“Accumulating data from all sources client received depot as prescribed on the below dates:…”*
13	N.1.2	In residential care	Monitored by facility in Aus. context		*“Lives in boarding house where medication is supervised”*
*32*	*A.1.2*	*Data gathered person-to-person*			
5	F.2.2	Client Confirmed			*“[DATE]: contact with client. confirmed compliance. no problems”*
14	F.2.3	Clinician or Case Manager Confirmed			*“Case manager: According to CPMS all medication has been dispensed for every month”*
4	F.2.6	Family confirmed			*“Client's mother pick-ups the medication and deliver at his place”*
2	F.3.2	Client lost to follow-up, case manager pursuing	Client disappeared, or disengaged		*“Client whereabouts unknown. Case manager aware of non-compliance”*
2	F.3.3	Client refused further intervention			*“P/C to client, declined services. Fax sent to GP.”*
1	F.3.4.2	Initiated with Medical professional support			*“Spoke to client, stopped antipsychotic as recommended by [psychiatrist]”*
3	F.3.4.3	Patient initiated, unsupported			*“Stopped taking medication as he had been on it for a long time (2 months) and it did not help.”*
2	N.2.4.1	Patient has case manager	Unclear if C.M. was followed up		*“Case managed, known non-compliance”*
123	A.2	Deferral to closer clinical contacts of the non-adherent person (Recency)			
14*	F.2.3	Clinician or Case Manager Confirmed			*“Case manager: According to CPMS all medication has been dispensed for every month”*
11	N.1.1	Changed Medication			*“Paliperidone injection monthly changed to 3-monthly (TRINZA)”*
15	N.1.5	Seen their GP, while			*“Seen GP since, assuming it was intended as CPZ was commenced since”*
13	N.1.5.1	Compliant with other medications			*“Regular in everything else, likely per Gp”*
2	N.1.5.2	Started a new medication			*“GP seemingly trying different antidepressants”*
68	N.2.2	GP visit regularity only source of data for assumption			*“Several GP appts since- assuming intentioned”*
166	A.3	Rules don’t always meet the contextual needs of prescribers and patients			
115	*A.3.1*	*Medications are prescribed and taken in more than one way*			
79	N.1.3	Likely PRN	PRN = taken as needed		*“PRN medication such as diazepam and oxacepan should not set triggers”*
22	N.1.4	Script likely a short-term solution			*“Was a once off script, regular GP visits”*
14	N.2.1	Assumed to be a trial			*“As only once dispensed I assume that it was poorly tolerated”*
51	*A.3.2*	*This style of follow-up is not always warranted or appropriate*			
42	N.2.3	Irregular prescription pickup pattern	Ie., assumed normal behaviour for pt		*“Likely timing issue, picked up the last repeat too early, long term very reliable”*
9	N.2.4	Patient known to be non-compliant, not followed up			*“Patient has a history of noncompliance with limited benefit of medication, as such likely real alert but no action taken before next scheduled review”*

#### A.1 Access to contextual information enables decision making

This theme contained two subthemes: A.1.1) Data gathered from other record-keeping systems; and A.1.2) Data gathered person-to-person. Beginning with the former, in 56 cases the screening clinician was able to determine the status of people flagged for non-adherence through querying other record-keeping systems. Regarding the other codes in this category, while medications and prescriptions issued in residential care, long acting injectables, with the support of a case manager, or as part of the clozapine protocol *would* be visible on PBS records as this version of AI^2^ operated on a weekly refresh it is reasonable that the clinician—upon confirming any of the latter—would not spend time following up on data that *may* be superceded by the next system refresh. In both cases, these insights would either have been requested from other systems and databases or noted within the trialling service’s clinical information system. Accessing these data constitutes a form of follow-up; while the person flagged as non-adherent was not directly contacted, non-AI^2^ data provided veracity for the clinician’s decision. This code also highlights the impact of the lack of integration within Australian contexts in which medical support is provided on attempts to monitor adherence and, indeed, on the maintenance of comprehensive records for people with complex interactions with health and carceral systems [73].

In 32 cases*, person-to-person data* (A1.2) was an important part of confirming adherence status. Sources included family, case managers, other clinicians, or the client themselves. This is important to note.

#### A.2 Deferral of action to closer clinical contacts of the non-adherent person

In 123 cases, the screening clinician deferred to the judgment of the clinician who most recently saw the person flagged as non-adherent. Most regularly cited were general practitioners, sometimes in combination with AI^2^ showing compliance with other medications (n = 13), or a new medication in place of the medication ceased (n = 2), but in the majority of cases with no other justification (n = 68).

#### A.3 Rules don’t always meet the contextual needs of prescribers and clients

This theme contained two subthemes: A.3.1) Medications are prescribed and taken in more than one way; and A.3.2) This style of follow-up is not always warranted or appropriate. To the former, people often take medications in patterns that differ from the most common use. Medication taken *pro re nata*—or, when needed—is course of action undertaken regularly in mental health services [74]. Additionally, changing dosage of a medication on a relatively fixed schedule—such as in some presentations of premenstrual dysphoric disorder [75]—also does not translate into a set-dose-per-day usage easily detected algorithmically. As such, the clinical monitor determined in 115 cases that the medication had been prescribed outside of the usecases monitored by the AI^2^ algorithm—but in line with what they might expect in practice for that drug. While any additional sources for making this determination were not cited in *any* of these cases, this code reinforces the potential utility of the data captured in A.1 for verifying these assertions.

In terms of the latter theme, in 51 cases the screening clinician made the determination that *this style of follow-up was not warranted or appropriate* (A.3.2). On the face of it, the two descriptive codes in this category contain radically different categories of risk—people known to by non-adherent, and those who pick up their prescriptions in an irregular manner. In terms of the latter, the labour costs involved in following up may outweigh the benefits. On the other hand, in the former, for repeatedly non-adherent clients a phone call to follow-up may be minimally impactful on their behaviour, or possibly adversely affect the therapeutic alliance with the service.

Mixed-Methods Analysis: Preliminary insights into client and medication subtype characteristics’ impact on follow-up behaviours.

### H1: Differences between medication subtypes and their likelihood to be followed-up

The proportion of flags that were not followed up and provided insufficient evidence, on review by the research team, to assume adherence (see Table 2) differed significantly between medication types (χ^2^ = 67.37; *p* < 0.001). Pair-wise Chi-squared tests between the four largest medication subtypes showed Anti-depressants were significantly less likely to be followed up than Antipsychotics (χ^2^ = 35.196, *p* < 0.001, v = 0.389), and Anxiolytics (χ^2^ = 44.825, *p* < 0.001, v = 0.499), but not Mood Stabilisers (χ^2^ = 1.455; *p* = 0.228). The other medication subtypes were excluded from this analysis due to their small sample sizes limiting reliable reporting of results (Tables 5, 6, 7).

**Table 5:** Medication type × follow-up status chi-squared test of homogeneity

Prescription Type	N. Flags	%	N. Followed-up (Expected Count)	N. Not Followed-up (Expected Count)
Mood stabilisers	21	6	5 (4.64)	16 (16.35)
Anti-psychotics	102	31	37 (22.55)	65 (79.45)
Anxiolytics	50	15	2 (11.06)	48 (38.94)
Anti-depressants	130	39	23 (28.76)	107 (101.25)
Totals	331		71	260
χ^2^ (p)				22.9121 (< .001)

As only one cell has an expected count < 5 (ie., 12.5% of cells), and χ^2^ > 7.82 (the critical χ^2^ value for tests with three degrees of freedom), therefore the assumptions of the test are met [88–90]

**Table 6:** Pairwise Fisher’s exact tests of independence for follow-up status between-medication subtypes

Prescription type	N. Flags	N. Followed-up (Expected Count)	N. Not Followed-up (Expected Count)
Mood stabilisers × Anti-psychotics			
Mood stabilisers	21	5 (4.64)	16 (16.35)
Anti-psychotics	102	37 (22.55)	65 (79.45)
*Totals:*	123	42	81
Fisher’s exact test	p = .322; no significant difference in follow-up status		
Mood stabilisers × Anxiolytics			
Mood stabilisers	21	5 (4.64)	16 (16.35)
Anxiolytics	50	2 (11.06)	48 (38.94)
*Totals:*	71	7	64
Fisher’s exact test	p = 0.021; significant difference in follow-up status		
χ^2^ (p; v)	4.49 (p = 0.0341; v = .303)		
Anxiolytics × Anti-psychotics			
Anxiolytics	50	2 (11.06)	48 (38.94)
Anti-psychotics	102	37 (22.55)	65 (79.45)
*Totals*	152	39	113
Fisher’s exact test:	*p* < 0.001; significant difference in follow-up status		
χ^2^ (p; v)	16.67 (p < 0.001; v = 0.341)		
Anti-depressants × Mood stabilisers			
Anti-depressants	130	23 (28.76)	107 (101.25)
Mood stabilisers	21	5 (4.64)	16 (16.35)
Totals:	151	28	123
Fisher’s exact test	p = 0.5463; no significant difference in follow-up status		
Anti-depressants × Anxiolytics			
Anti-depressants	130	23 (28.76)	107 (101.25)
Anxiolytics	50	2 (11.06)	48 (38.94)
*Totals:*	180	25	155
Fisher’s exact test:	p = 0.016; significant difference in follow-up status		
χ^2^ (p; v)	5.66 (p = 0.0174; v = 0.1773)		
Anti-depressants × Anti-psychotics			
Anti-depressants	130	23 (28.76)	107 (101.25)
Anti-psychotics	102	37 (22.55)	65 (79.45)
*Totals:*	232	60	172
Fisher’s exact test:	p = 0.0015; significant difference in follow-up status		
χ^2^ (p; v)	10.29 (p = 0.0013; v = 0.2106)

**Table 7:** Normality of distributions—days to action × medication subtypes

Distribution	M (SD, 95% CI)	Shapiro–Wilk tests of normality		
		Statistic	df	Significance
Days to action × Anti-epileptics	5.000 (2.302, 3.952–6.047)	.916	21	0.071
Days to action × Anti-parkinsonians	3.667 (2.065, 1.499–5.834)	.917	6	0.487
Days to action × Anti-psychotics	4.637 (2.331, 4.183–5.091)	.938	102	< 0.001
Days to action × Anxiolytics	3.800 (2.050, 3.217–4.824)	.849	50	< 0.001
Days to action × Sedatives	3.000 (2.121, 0.336–5.634)	.899	5	0.405
Days to action × Anti-depressants	4.369 (2.382, 3.956–4.783)	.932	130	< 0.001
Days to action × Psychostimulants	5.833 (2.125, 4.483–7.183)	.971	12	0.918
Days to action × Nervous system drugs	4.200 (2.683, 0.868–7.532)	.838	5	0.160

## H2: Differences between medication subtypes and timeliness of follow-up

The distributions associated with Days to Action x Medication Subcategory were not all normally distributed. A Kruskal–Wallis H-Test showed no significant differences in the distribution of days to respond between medication subtypes (H = 12.825; p = 0.077).

## H3. Differences in time-to-action between follow-up categories.

**Fig. 5:**
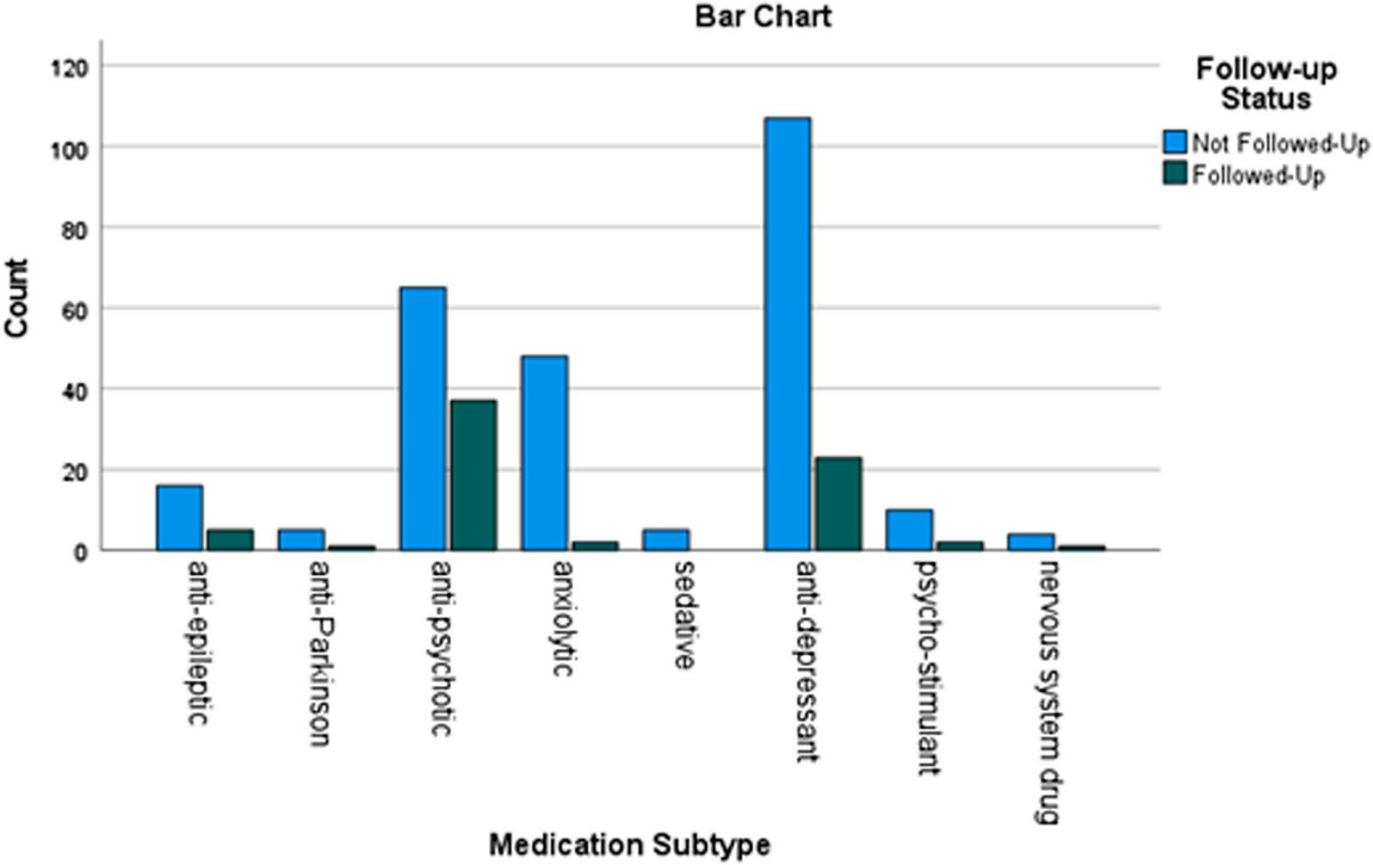
Follow up status × Days to Action

The days taken to action Not Followed-up(0) and Followed-up(1) flags were compared (Fig. 5). The normality of the distributions was tested using Shapiro–Wilk tests of Normality, which showed significant deviance from normality (p_0_ < 0.001; p_1_ = 0.026). A Mann–Whitney U test found a significant difference, however with a modest effect size, between the distributions of response times between follow-up categories (M_0_ = 31.78; Range_0_ = 116; IQR_0_ = 38; M_1_ = 45.55; Range_1_ = 129; IQR_1_ = 30; U = 6341; *p* < 0.001; Z = 4.043; η^2^ = 0.05).

## H4: Time × Event differences within-clients with mixed-follow up status flags

**Fig. 6:**
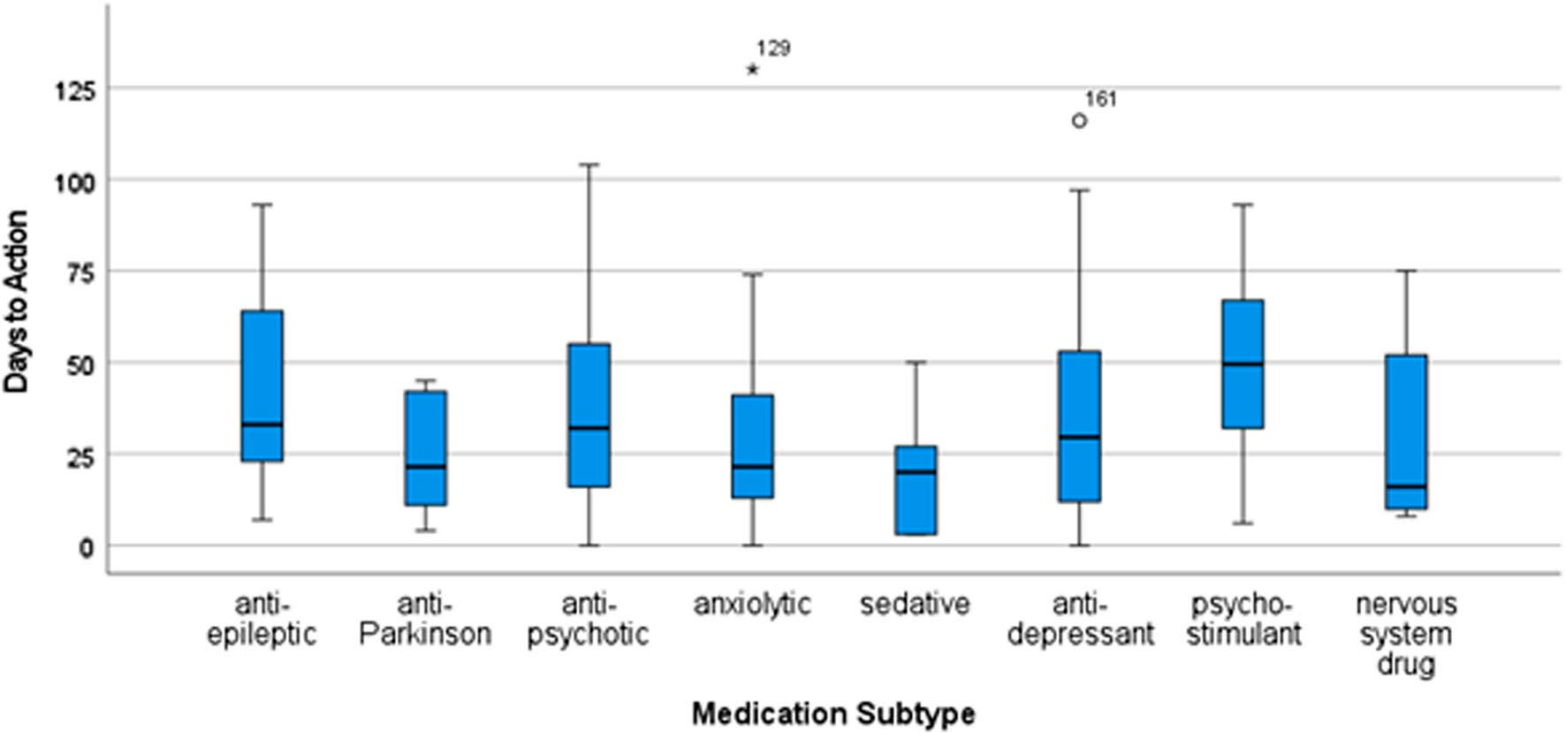
Frequency of number of flags per client

Data for 179 clients was included in this analysis. Most clients were flagged once, although this varied up to six flags for some (Fig. 6). 39 clients were exclusively followed up, 133 clients were exclusively not followed up, and 9 clients had flags in both categories. These data were insufficient for further quantitative analysis.

## Discussion

This study provides insights into CDSS design and clinician behaviours, from which researchers and services can derive sites of intervention to better improve to medication guidelines in real-world clinical mental health services.

## Summary of findings

The majority of clients who were flagged were not followed-up. In those that were, qualitative analysis showed that contextual information enabled decision making. Where there was no follow-up, there was a tendency to defer—where contact had been made recently—to the judgment and monitoring of more recent clinical contacts (usually primary care) of the client. Additionally, the clinical monitors in many cases determined that either the rules of the algorithm or the intervention itself did not meet the context of the client. These findings indicate, overall, that contextually aware CDSS designs—that is, design that can take into account the person’s environment, clinical relationships and medical needs, executed with or without automation—show potential for enabling naturalistic follow-up interventions.

These qualitative findings were further elaborated by the mixed-methods results, which indicate—preliminarily—that time and effort costs associated with following up lower-risk non-adherence events (such as anti-depressants) may be perceived to outweigh the benefits (H1). Additionally, the quantitative results indicate more broadly the lack of faith in the veracity of prompts to follow-up generated from EHR data alone; indeed, the finding that Followed-up flags took significantly longer than Not Followed-Up flags may indicate that more clear-cut non-adherence data were a key ingredient, at least in this trial, for encouraging action (H3). Finally, that there were insufficient data to test within-client changes in follow-up status (H4) indicates the complexities of what repeated non-adherence—either actual, or as an artefact of algorithms—can represent. Indeed, these findings further affirm the Thematic Synthesis finding that the inflexibility of algorithmic “rule-breaking” inherently produces a reliance on clinical judgment of non-adherence, the general lack of veracity indicated in both the thematic synthesis and results for H3, and the importance of integrating non-EHR data into CDSS to address these limitations.

### Implications for further research

#### Encouraging client and caregiver engagement and autonomy

The majority of clients who were flagged for medication non-adherence in this study were not followed up, with a significant lack of follow-up for anti-depressant non-adherence (H1)—a class of drugs, as established in the background to this study, prescribed for many conditions [76, 77], with potentially severe discontinuation effects [18], but considered low risk due to both their sometimes short-term use and guidelines indicating minimal discontinuation effects [17]. Regardless of risk, these clients are difficult to identify in the data currently collected by AI^2^, may have severed their relationship with their clinician [13, 22, 78], and the costs (time and effort) associated with the current intervention may outweigh the impact on the possibly small proportion of people who would benefit—an assertion backed by Analytical Themes A.1 and A.3. In the context of the finding that followed-up flags took significantly longer to action (H3)—indicating that a longer period of time since the flag was first raised and, therefore, a more clear-cut indication of non-adherence gave clinicians more impetus to act—it is further indicated that follow-up, if it were to happen, would likely happen outside of the window where discontinuation effects and/or encouraging restarting medication were feasible outcomes. Analytical Theme A.1 offers an inroad for design insights into these findings which, when synthesised, highlight a need for increased *veracity* of data within the CDSS—which A.1 indicates may be achieved through the incorporation of different data streams between both different record-keeping systems and between human actors.

One avenue of achieving this is through incorporating clients and their caregivers as both empowered actors and data sources within systems. Indeed, a systematic review found CDSS studies that incorporated input and follow-up from clients and caregivers to be more effective, potentially through the empowering, engaging and, therefore, clinician accountability building effect handing consumers these data can have [68]. If well designed and implemented, these methods also have the potential to provide more actionable insights into the experiences of people who abruptly discontinue “lower-risk” drugs, such as anti-depressants [31, 42, 79]; addressing the cost–benefit dilemma of the current intervention. Finally, this approach could also be utilised in clients who identify themselves as struggling with adherence to provide motivational, health-promoting, or supportive content— an important and potentially efficacious adjunct for this group [79, 80].

#### Interoperability with, or automated data collection and follow-up between services and systems

Adherence is not a monolithic category [1]. Non-adherence can appear as (a) clients simply not picking up a prescription (non-fulfilment); (b) clients can pick up a prescription, but then stop taking medication after initially taking it (non-persistence)—which can be both deliberate or due to lack of capacity or resources on the client’s part; and c) taking medication, but not in the manner in which it was prescribed (non-conforming) [1, 4]. Considering that: all three of these categories can occur simultaneously with the client not informing their GP or clinician of their non-adherence, low adherence among clients with chronic and complex conditions to *all* of their prescribed medications within complex drug regimes [7, 81–83], the finding in A.2 that clinical monitors working with AI^2^ had a tendency to defer to closer clinical contacts of the person flagged as non-adherence, the volume of Not Followed-Up flags categorised in the Framework analysis as Unclear Without Further Investigation—it is clear that further development and evaluation of communications between clinical monitors and other clinicians involved in the care of people flagged by AI^2^ is necessary.

Indeed, adherence approaches at their best are collaborative [1, 4]—between clinicians, services, and clients—and the development of automated notification and data collection systems for further implementations should, therefore, also aim to integrate data *from* and follow-up *with* other services and systems. Data collected *from* other systems, services and clinicians could be feasibly extracted from other, yet-to-be-implemented areas of MyHealthRecord—such as prescription and dispense records, shared health summaries, and event summaries—using techniques such as natural language processing, or careful presentation of raw data to enable further insights. Additionally, automated email contact initiations could be utilised. Automated follow-up of other services, systems and clinicians could involve interventions such as prompting clinicians—via email or other methods—to consider the potential impacts of different follow-up paths or, more simply, reminding the clinician of the value of the intervention [84]—design patterns from other industries that have been suggested as being potentially applicable to health [61].

#### The continuing importance of human factors

Additional to our provocations to consider automated follow-up, it is important to continue to stress the contextual and human factors within-services that facilitate or block CDSS use [50, 53, 61]. This study provides a nuanced set of initial insights, using novel data, to the interaction design literature seeking to address this.

First, in all clinical decision support systems it is necessary to balance the impulse to notify against the actionability of the notification, both to avoid alert fatigue and minimise the risk of adverse outcomes or legal ramifications [69, 70, 85]. In the context of these difficult—or unnecessary—to action flags, the use of filters in AI^2^ and similar systems could be used to narrow the use case to target specific client groups—allowing for a greater sense of specificity, the optimisation of which may facilitate adherence to CDSS use [69]. For example, in this study site focussing on follow-up of anti-psychotics may have been preferable when considering, in hindsight, the quantity of data generated by AI^2^ and the service’s priorities for follow-up. This methodology provides an option for clinics to ease into proactive care while balancing existing duties—or scoping the resources required to expand coverage as they arise. Combined with other methods of automated follow-up, this may improve timeliness, clinician workload concerns, as well as client and clinician outcomes more broadly.

### Limitations

This study demonstrates the potential of the Medicare data for monitoring and following up on non-adherence. These data do not include services sought from private mental healthcare. However, because of the often chronic and high-cost nature of living with a mental health condition in Australia, Medicare funded services are widely utilised by people with mental illnesses in Australia. Additionally, while Medicare allows state based acute services to view federally regulated and funded GP activities and occasions of pathology, radiology, and so on, acute care services funded by state government like hospitals are not visible in this data. Finally, as these are pilot findings—collected from a small number of clinicians and analysed by a single coder—their generalisability should be considered cautiously.

## Conclusions

This study highlights the interaction design challenges facing health services and researchers implementing proactive care processes using CDSS. In particular, these results point towards the importance of addressing perceptions of: (1) risks associated with non-adherence to different medication-types; (2) the veracity of non-adherence data provided by CDSS; and (3) the person’s environment, clinical relationships and medical needs, and how associated biases related to their adherence. We suggest the importance of considering context in increasingly automated follow-up interventions as a priority for future research.

## Supplementary Information

**Additional file 1**. Appendix one: APA MMARS Reporting Standards Conformance Table.

## Data Availability

The datasets generated and/or analysed during the current study are not publicly available due to the potentially identifying information contained within, due to the study’s location in a small community. There data can, however, be available from the corresponding author on reasonable request and after consultation with the Southern Adelaide Local Health Network Clinical Research Ethics Committee.

## Abbreviations

CDSS: Clinical decision support system(s)
AI^2^: The Actionable Intime Insights study and software [30]
H(n): Hypothesis (n)
MyHR: My Health Record, the Australian National eHealth Record
PRN: As needed (Latin: *Pro Re Nata*)
RCT(s): Randomised Control Trial(s)
EHR: Electronic Health Record(s)
CIS: Clinical Information System(s)
