# Supplementary material for: Examining clinician choice to follow-up (or not) on automated notifications of medication non-adherence by clinical decision support systems
Source: BMC Med Inform Decis Mak. 2023 Jan 30;23:22. doi: 10.1186/s12911-022-02091-2 (PMC9887874; doi:10.1186/s12911-022-02091-2)
Supplement: Supplementary file 1 — Additional file 1. Appendix one: APA MMARS Reporting Standards Conformance Table. [file 12911_2022_2091_MOESM1_ESM.docx]

# APPENDIX ONE: APA-JARS: MMARS. Reporting Standards Conformance Table.

| Item | Guideline | Example in-text/Section (page/line) |
| --- | --- | --- |
| Title | Identify main variables and theoretical issues under investigation and the relationships between them. • Identify the populations studied.  Guidance for Authors ‒ Refrain from using words that are either qualitative (e.g., "explore," "understand") or quantitative (e.g., "determinants," "correlates"), because mixed methods stands in the middle between qualitative and quantitative research. ‒ Reference the mixed methods, qualitative methods, and quantitative methods used. | Examining in-the-moment clinician decisions to follow-up (or not) on automated medication non-adherence alerts: A Mixed-Methods Analysis |
| Author Note | -Acknowledge funding sources or contributors.  -Acknowledge conflicts of interest, if any  Provide acknowledgment and explanation of any special circumstances, including:  ‒ registration information if the study has been registered  ‒ use of data also appearing in previous publications  ‒ prior reporting of the fundamental data in dissertations or conference papers  ‒ previous (or current) affiliation of authors if different from location where the study was conducted  ‒ contact information for the corresponding author  ‒ additional information of importance to the reader that may not be appropriately included in other sections of the paper | Declaration  Declaration  Abstract; Declaration  Introduction; Methods  As above.  N/A  Abstract  Methods |
| Abstract | State the problem/question/objectives under investigation.  Indicate the mixed methods design, including:  -types of participants or data sources,    -analytic strategy,  -main results/findings, and  -major implications/significance.  Guidance for Authors  ‒ Specify the type of mixed methods design used.  ‒ Consider using one keyword that describes the type of mixed methods design and one that describes the problem addressed.  ‒ Describe your approach(es) to inquiry and, if relevant, how intersecting approaches to inquiry are combined when this description will facilitate the review process and intelligibility of your paper. | Abstract: Background  Abstract: “These data were collected in parallel, embedded within the pilot implementation”  This study examines patterns in clinician decision making behaviour. Flags related to medication non-adherence with deidentified clinical decision note data were included for analysis.  Notes were described and then analysed using thematic synthesis  Abstract: Results  Abstract: Conclusion  Embedded.  Keywords  Abstract: Methods |
| Introduction | **Problem**  • Frame the problem or question and its context.  • State the importance of the problem, including theoretical or practical implications.  **Review of Relevant Scholarship**  • Review, critique, and synthesize the applicable literature to identify key issues/debates/ theoretical frameworks in the relevant literature to clarify barriers, knowledge gaps, or practical needs  ‒ relation to previous work  ‒ differences between the current report and earlier reports if some aspects of this study have been reported on previously Hypothesis, Aims, and Objectives  **Hypothesis, Aims and Objectives**  • State specific hypotheses, aims, and objectives, including ‒ theories or other means used to derive hypotheses ‒ primary and secondary hypotheses ‒ other planned analyses  • State how hypotheses and research design relate to one another  State the purpose(s)/goal(s)/aim(s) of the study.  • State the target audience, if specific.  • Provide the rationale for fit of design used to investigate this purpose/goal (e.g., theory building, explanatory, developing understanding, social action, description, highlighting social practices).  • Describe the approach to inquiry, if it illuminates the objectives and research rationale (e.g., descriptive, interpretive, feminist, psychoanalytic, postpositivist, critical, postmodern, constructivist, or pragmatic approaches).  Guidance for Authors ‒ If relevant to objectives, explain the relation of the current analysis to prior articles/ publications.   - State three types of research objectives/aims/goals: qualitative, quantitative, and mixed methods. Order these goals to reflect the type of mixed methods design used. - Describe the ways approaches to inquiry were combined, as it illuminates the objectives and mixed methods rationale (e.g., descriptive, interpretive, feminist, psychoanalytic, postpositivist, critical, postmodern, constructivist, or pragmatic approaches). | Background: The Current Study  Background  Background  Background  Aims stated at end of background; futher elaborated in Table 1 of Methods.  Methods; Table 1 and Theoretical Background to Current Study.  N/A  Background: The Current Study  Methods: Theoretical background  Methods: Prior Trial Details  Methods: Table 1; end of Background  Background; Methods: Theoretical Background |
| Methods | Inclusion and Exclusion  • Report inclusion and exclusion criteria, including any restrictions based on demographic characteristics.  Participant Characteristics  • Report major demographic characteristics (e.g., age, sex, ethnicity, socioeconomic status) and important topic-specific characteristics (e.g., achievement level in studies of educational interventions).  • Explain why mixed methods research is appropriate as a methodology given the paper’s goals  .  • Identify the type of mixed methods design used and define it.  • Indicate the qualitative approach to inquiry and the quantitative design used within the mixed methods design type (e.g., ethnography, randomized experiment).  Sampling Procedures  • Describe procedures for selecting data, including ‒ sampling method if a systematic sampling plan was implemented ‒ percentage of sample approached that actually participated ‒ whether self-selection into the study occurred (either by individuals or by units, such as schools or clinics)  • Describe settings and locations where data were collected as well as dates of data collection.  • Describe agreements and payments made to participants.  • Describe institutional review board agreements, ethical standards met, and safety monitoring.  Data Collection  • Describe methods used to collect data.  • If multiple approaches to inquiry were combined, describe how this was done and provide  a rationale (e.g., descriptive, interpretive, feminist, psychoanalytic, postpositivist, critical,  postmodern, constructivist, or pragmatic approaches), as it is illuminating for the mixed method in use.  • Provide a rationale or justification for the need to collect both qualitative and quantitative  data and the added value of integrating the results (findings) from the two databases.  Analytic Strategy  • Describe the analytic strategy for inferential statistics  •When data are collected from multiple sources, clearly identify the sources of qualitative and quantitative data (e.g., participants, text), their characteristics, an paragraph about the relationship between the data sets, if there is one (e.g., an embedded design).  • State the data sources in the order of procedures used in the design type (e.g., qualitative sources first in an exploratory sequential design followed by quantitative sources), if a sequenced design is used in the mixed methods study  • Indicate methodological integrity, quantitative validity and reliability, and mixed methods validity or legitimacy. Further assessments of mixed methods integrity are also indicated to show the quality of the research process and the inferences drawn from the intersection of the quantitative and qualitative data. | Methods: The Current Study  Methods: Prior Trial Details  Methods: Inferential Analyses  Methods: Theoretical Background. Background: End.  Methods: Analysis Plan  Methods: The Current Study  Methods: Prior Trial Detials  N/A (part of usual care)  Methods: Ethics  Methods: Data Collection  Methods: Theoretical Background  Methods: Inferential Analyses  Methods: Inferential Analyses; Table 1  N/A  Methods: Procedure  Methods: Risk of Bias |
| Results | Indicate how the qualitative and quantitative results were “mixed” or integrated (e.g., discussion; tables of joint displays; graphs; data transformation in which one form of data is transformed to the other, such as qualitative text, codes, themes are transformed into quantitative counts or variables).  • Describe research findings (e.g., themes, categories, narratives) and the meaning and understandings that the researcher has derived from the data analysis.  • Demonstrate the analytic process of reaching findings (e.g., quotes, excerpts of data).  • Present research findings in a way that is compatible with the study design.  • Present synthesizing illustrations (e.g., diagrams, tables, models), if useful in organizing and conveying findings.  ‒ descriptions of each primary and secondary outcome, including the total sample and each subgroup, that includes the number of cases, cell means, standard deviations, and other measures that characterize the data used  ‒ inferential statistics, including › results of all inferential tests conducted, including exact p values if null hypothesis significance testing (NHST) methods were used, and reporting the minimally sufficient set of statistics (e.g., dfs, mean square [MS] effect, MS error) needed to construct the tests › effect-size estimates and confidence intervals on estimates that correspond to each inferential test conducted, when possible › clear differentiation between primary hypotheses and their tests–estimates, secondary hypotheses and their tests–estimates, and exploratory hypotheses and their test–estimates  • Report any problems with statistical assumptions and/or data distributions that could affect the validity of findings | Tables 2, 3, and 4.  Tables 2, 3, and 4. Results.  Methods: Analysis Plan.  Results. Tables 2, 3, and 4.  Tables 2, 3, and 4.  Tables 2,3 & 4; Figs. 5 onwards.  Results: Mixed-methods analysis.  Insufficient data for H4 |
| Discussion | •Provide a statement of support or nonsupport for all hypotheses, whether primary or secondary, including ‒ distinction by primary and secondary hypotheses ‒ discussion of the implications of exploratory analyses in terms of both substantive findings and error rates that may be uncontrolled  • Describe the central contributions and their significance in advancing disciplinary understandings.  • Describe the types of contributions made by findings (e.g., challenging, elaborating on, and supporting prior research or theory in the literature describing the relevance) and how findings can be best utilized.  • Identify similarities and differences from prior theories and research findings.  • Reflect on any alternative explanations of the findings.  • Identify the study’s strengths and limitations (e.g., consider how the quality, source, or types of the data or the analytic processes might support or weaken its methodological integrity).  • Describe the limits of the scope of transferability (e.g., what should readers bear in mind when using findings across contexts).  • Revisit any ethical dilemmas or challenges that were encountered, and provide related suggestions for future researchers.  • Consider the implications for future research, policy, or practice  • Discuss generalizability (external validity) of the findings, taking into account ‒ target population (sampling validity) ‒ other contextual issues (setting, measurement, time; ecological validity) | Discussion: Summary of Findings  Discussion  Discussion: Implications  Discussion: Implications  Limitations  Discussion; Limitations  Limitations  N/A  Discussion; Implications  Discussion; Implications |
